# Exploring Novel Fungal Bioremediation Treatments to Inhibit Pollutants and Microbial Hazards Associated with Untreated Biological Soil Amendments of Animal Origin

**DOI:** 10.3390/microorganisms13122847

**Published:** 2025-12-15

**Authors:** Alexis N. Omar, Anastasia E. M. Chirnside, Kalmia E. Kniel

**Affiliations:** 1 Department of Animal and Food Sciences, University of Delaware, 531 S. College Avenue, Newark, DE 19716, USA; 2 Department of Entomology and Wildlife Ecology, University of Delaware, 531 S. College Avenue, Newark, DE 19716, USA

**Keywords:** mycoremediation, mycofiltration, bioremediation, biological soil amendments of animal origin (BSAAOs), windborne transmission, white-rot fungi

## Abstract

Biological soil amendments of animal origin (BSAAOs) provide risk for foodborne contamination. Soils are often enriched with BSAAOs to increase nutrient value, enhance and support crop growth and yield. Little is known about the interactions of soil microorganisms and the potential impact on food safety. Although BSAAOs provide benefits to soil and crops, BSAAOs are a risk for contamination. Another source of risk includes adjacent land use of concentrated animal feed operations (CAFOs) and the risk of contaminated dust with pathogens such as *Escherichia coli* or *Salmonella* is becoming more of a concern. Studies have shown that crops planted adjacent to a cattle feedlot were contaminated with pathogenic *E. coli* O157:H7 which originated from the cattle feedlot. Further research is needed to evaluate novel bioremediation techniques to lower/prevent the risks of windborne contamination of dust and risks posed by untreated BSAAOs. One potential novel technique is the utilization of mycofiltration. The risks of pathogenic contamination of BSAAOs could be reduced by developing a cost-effective and sustainable mycofiltration practice using naturally formulated by-products from filamentous fungi. Ligninolytic white-rot fungi can degrade a wide variety of toxic or persistent environmental contaminants and degrade pollutants in the environment. Recent studies have shown that white-rot fungi can inhibit pathogenic *E. coli* in bioreactor systems. Exploring white-rot fungi as a biocontrol agent for on-farm mycofiltration may prove to be a cost-effective treatment and limit certain routes of contamination to the edible portion of the crop, certainly worthy of exploration in this review.

## 1. Introduction

Pathogenic contamination of biological soil amendments, such as untreated poultry litter and dairy manure, can have impactful health and economic effects. Health concerns of contaminated soil can include the presence of foodborne pathogens such as Escherichia coli O157:H7 and Salmonella enterica subsp. as well as others [1]. In the United States, there are an estimated 48 million illnesses, 128,000 hospitalizations, and 3000 deaths due to foodborne diseases each year [2]. It is projected that, in the U.S, foodborne illnesses cost an estimated USD 255 million annually, contracted from the bacterial pathogen *E. coli* O157:H7 alone [3]. Foodborne diseases also cause a substantial public health, economic, and social burden globally [4]. The World Health Organization (WHO) reported the first estimates of global and reginal disease burden due to 31 foodborne hazards [4]. Results showed that each year, 1 out of 10 people globally will become ill from foodborne diseases, resulting in 600 million illnesses, 420,000 deaths, and the loss of 33 million healthy years of life globally [4]. Pathogenic bacteria are risks to the food safety of raw agricultural crops, as well as to the health and safety of growers. Runoff from these amended agricultural lands also carries the risk of polluting natural bodies of water within the surrounding area [5]. With the increased demand for organic farming [6] and the increased use of biological soil amendments of animal origins (BSAAOs) [7], there is a need to identify alternative methods to decrease bacterial contamination that are also compatible with the USDA’s National Organic Program.

The objectives of this review are to evaluate the risks of pathogenic contamination associated with BSAAOs and describe how novel mycoremediation approaches may be the key to combating contamination. Drawing upon several peer-reviewed articles and comprehensive reviews published between the years 1998 and2025, this review aims to synthesize evidence across the mid-Atlantic region of the United States to thereby delineate the global scope and reginal specificities of this issue.

## 2. Biological Soil Amendments of Animal Origin

As defined by the U.S Food and Drug Administration (FDA), a BSAAO is a biological soil amendment that consists of materials of animal origin, such as manure or non-fecal animal byproducts [8]. BSAAOs include manure, compost, and compost teas that are frequently used in conventional and organic agricultural practices, which prohibits farmers from using synthetic fertilizers and pesticides [9,10]. In accordance with the Produce Safety Rule, Subpart F, a BSAAO is defined as untreated if it meets any of the following conditions: (a) it has not been processed to completion in accordance with the requirements of the Produce Safety Rule subpart F 112.54; (b) it has become contaminated after treatment; (c) it has been recombined with an untreated BSAAO; or (d) it is, or contains, a component that is untreated waste that you know or have reason to believe is contaminated with a hazard or has been associated with a foodborne illness [8]. The most commonly used untreated BSAAOs include cattle manure, poultry litter, swine slurry, and horse manure [8]. Using untreated BSAAOs can pose unprecedented risks of contamination and risks of foodborne illness and outbreaks. Current language in the Food Safety Modernization Act Produce Safety Rule states no objections to a 90- or 120-day interval between the application of untreated BSAAOs and the harvest of crops to minimize transfer of produce intended for human consumption with the intent to help limit potential cases of foodborne illness [11]; however, studies have shown that *E. coli* species can persist past the 90- or 120-day time interval from the application of untreated BSAAOs to harvest, due to specific regional spatiotemporal effects [5].

Several treatment methods are used to reduce the risk of contamination through the inactivation of foodborne pathogens in manure. The treatment methods that are commonly used can be classified into three types: physical, chemical, and biological treatments [12,13]. Several studies have investigated these three treatment types, and they report variable reductions and survival of bacterial and viral species after treatments, as shown in Table 1.

### 2.1. Physical Heat Treatment of Animal Manure

Physical treatment of manure utilizes heat and irradiation to inactivate pathogens. Pelletization, also known as granulating manure, is a method of agglomerating manure into a dry product known as granules, pellets, or agglomerates [22]. The process can involve using different techniques, such as extruders, pan, or disk granulators, pin mixers, or spray congealing [23]. The particle size of the pellets can be adjusted by changing the mold size, mixing duration, pressure, and type of binder used [23]. The type of manure can also affect the best method for granulation.

Heat-treated poultry pellets (HTPPs) are a nutrient-rich organic fertilizer made from poultry manure that has been dehydrated and pelletized [15]. The process involves loading dry manure into a hopper, where it is chopped and pressed into pellets, then heat-treated at over 70 °C for an hour to inhibit pathogens [22]. The pellets are typically 5 mm in diameter and 20–30 mm long, but the size can be adjusted [22]. HTPPs are often used by fruit and vegetable growers as a slow-release fertilizer that can help improve soil health and crop production [24,25]. However, contamination of soil on farms may occur through contaminated irrigation water or scat from wild animals [24]. A study by Shah et al. [14] showed that the presence of HTPPs in soil led to a longer survival duration of *Salmonella* Newport in soil and to a greater likelihood of its transfer and survival on spinach plants [14]. Similarly, Litt et al. [5] showed that *E. coli* survival persisted past 120 days in soils amended with HTPPs compared to those without, indicating an increased risk of pathogen *E. coli* transfer to cucumber, spinach, and radish plants. Limoges et al. [15] evaluated soils across the Northeastern U.S. and the differential survival of *E. coli* and *Listeria* spp. on soils amended with dairy manure compost, poultry litter composter, and HTPPs and the fate of raw edible radish crops. The results were consistent with findings from studies conducted in other regions of the United States, which suggests that composted and non-composted poultry-based BSAAOs support greater survival of *E. coli* in field soils [15]. Shiga toxin-producing *E. coli* causes many outbreaks related to produce, and this research suggests that alternative practices when harvesting produce intended for raw consumption should be considered [15].

### 2.2. Chemical Treatment of Animal Manure

Chemical treatments utilize different chemical substances to be added to manure or manure slurry in concentrations lethal enough to inactive pathogens. Different kinds of chemicals that are normally used for chemical treatments of manure include formalin, sodium hydroxide, and 40% lime—wash, peracetic acid, calcium cyanamide, and a 50% caustic soda [13]. For the chemical treatment to be effective, the manure slurry needs to be thoroughly mixed prior to adding the disinfectants, this ensures that the concentration of chemicals is distributed equally across the homogenized manure slurry, and that there is sufficient contact time to ensure pathogen decontamination.

The best management practices for pathogen control in manure management systems continue to be a concern. One study on swine slurry and the effects of ozonation reported a 3 log reduction in *E. coli* and a 1 log reduction in total coliforms when the manure was treated with 2.0 g/L and 1.0 g/L of ozone [16]. No further reduction in *E. coli* or coliforms was observed with additional increased treatments of ozone [16]; however, ozone’s effectiveness is often inhibited by the high concentrations of organics present in the manure, thus requiring an additional solid separation pretreatment step [26].

A study conducted in Santa Catarina, Brazil evaluated the effectiveness of chemical treatments, using calcium hydroxide, on inactivating pathogens within wastewater from a swine manure treatment system [17]. It was found that after 24 h of chemical treatment, there was a complete inactivation of *E. coli* and *Salmonella* species [17]; however, the use of a chemical treatment is highly labor-intensive as it requires thorough mixing of materials. Another study by Decrey et al. [18] used Ammonia (NH_3_) concentrations (0 to 40 mmol L^−1^) as an in situ sanitizer to inactivate viruses in human excreta and animal manure (HEAM). DNA and double-stranded RNA (dsRNA) viruses were considerably more resistant than single-stranded RNA (ssRNA) viruses, resulting in an up to 1000-fold-longer treatment time to reach a 4 log inactivation. The slower inactivation of DNA viruses was rationalized by the higher stability of DNA than that of ssRNA in HEAM. Increasing the pH and temperature, such as those encountered in thermophilic digestion and alkaline treatments, leads to more consistent inactivation kinetics among ssRNA and other viruses, which suggests that the dependence of inactivation on genome type disappeared in favor of protein-mediated inactivation mechanisms common to all viruses [18]. In a related study, Wei et al. [27] determined that hepatitis A virus (HAV) and murine norovirus (MNV) were inactivated rapidly in alkaline pH biosolids and were unstable in liquid dairy manure (DM). In this study, MNV and HAV were inoculated into different types of animal manure and three types of differently treated biosolids at 20 °C and 4 °C for up to 60 days. Both viruses were inactivated rapidly in lime-stabilized biosolids with alkaline pH and were unstable in DM; however, when alum was added to poultry litter, it had different effects on the two viruses. Alum inactivated some HAV at both temperatures (20 °C and 4 °C) after 60 days but had no effect on MNV. This indicates that the application of some soil amendments can pose a risk to food safety and human health [27]; however, it also is important to note that virus stability and infectivity is dependent on external factors such as the virus, manure, and biosolid type [27].

Although the use of chemical treatment regimens has been proven very effective in reducing and inactivating pathogens within wastewater and manure slurries, it poses difficulties in instances where large volumes of chemicals are required. The increase in chemical volume can pose risks of health and safety to those involved in the process, as well as potentially endangering animals and the environment [28]. Therefore, it is best practice that the use of a chemical treatment to treat manure only be used in the case of an epidemic or instance of exemplary need [13].

### 2.3. Biological Heat Treatment of Animal Manure

Biological treatment utilizes substances of living organisms to treat and purify manure. When using biological treatment, there are also aerobic processes involved. The aerobic biological treatment process utilizes bacteria and oxygen, perhaps from injected air used to remove dissolved organic load such as chemical oxygen demand (COD) and biological oxygen demand (BOD) from wastewater or manure [29]. The aeration process of manure can occur in two different systems: natural aeration and mechanical aeration [29]. Natural aeration occurs when the manure is exposed to natural air and oxygen within a large holding tank with a depth of 5 ft to ensure the removal of pathogens. Mechanical aeration occurs by pumping oxygen, through the use of mechanical aerators, or by using both of these processes along with an injection of compressed air [26,28]. The use of aeration processes on manure or manure slurry at mesophilic temperatures is proven to be effective in reducing pathogens in instances where there is no fresh manure or manure slurry being added to the holding tank during the aeration process, which is typically 3–4 weeks [30]. The process of Aerobic Thermophilic Stabilization (ATS) uses the combined force of aeration and high temperatures of 50 °C to inhibit pathogens such as *Salmonella* and *E. coli* in cattle manure within 24 h [31]. One drawback of using an aeration system for pathogen removal is that its efficiency in removing pathogens depends on the weather conditions of the region. The system relies heavily on the temperature of the region to achieve high temperatures of 50 °C to inhibit pathogens, making it more effective in hotter climates and essentially ineffective in cooler climates [31]. Supplemental heat can be used to achieve high temperatures in cooler climates. However, due to the costly nature of supplemental heating and reduced effectiveness of aeration in cooler climates, they are not commonly used in cooler areas [26].

The antimicrobial effectiveness of anaerobic biological treatment of manure can reduce the number of indicator organisms but not eliminate them completely. In a study by Pourcher et al. [19] on swine manure, *Salmonella* spp. was detected in 60% of 17 raw manure samples and in 20% of the 10 treated manure samples. *Listeria* spp. was detected in 50% of the anaerobically stored samples and 20% in the samples which were treated anaerobically after aerobic digestion. Though there was a reduction in the number of pathogens and fecal indicators after aerobic digestion and the anaerobic storage treatment, pathogens were found in the manure up to the time of application [19]. Another study was conducted to determine the ability of an ecological treatment system to reduce the concentration of total coliform and *E. coli* from dairy wastewater by passing the wastewater through a series of anaerobic, aerobic, and clarifier reactors and wetland cells before leaving the system [20]. The indicator bacteria were consistently reduced by 99%, with a major reduction of 76% occurring within the first two reactors [20]. However, the *E. coli* numbers in the effluent did not meet the U.S. Environmental Protection Agency’s discharge requirements [20].

There is a need for a new research approach utilizing novel biocontrol agents to biologically treat manure. A system that has been studied to some degree that could improve the efficiency of current systems is the inclusion of a fungal bioreactor-based system. Taylor et al. [21] determined that fungal mycelium can act as a barrier or filter to inhibit the pathogenic bacteria from wastewater or the manure running through it. The use of filamentous fungi within a bioreactor system allows the fungi to utilize their mycelial mats, which can remove pollutants within wastewater or aqueous dairy manure [21]. Another study by Větrovský et al. [32] determined that white-rot fungus is able to utilize a wider range of extracellular enzymes, which allows it to be more suitable for mycofiltration remediation applications. Further research should study fungal-based mycoremediation use as a biocontrol pre-harvest preventative measure in biological soil amendments and within on-farm sanitation practices for untreated BSAAOs.

## 3. Pathogen Survival in BSAAOs

BSAAOs are well-studied regarding their potential as a risk for foodborne contamination. Soils are often enriched with BSAAOs to increase nutrient value, enhance the soil’s water-holding capacity, and support crop growth and yield [9]. Livestock production and the generation of BSAAOs are critical to sustainable food systems. According to the Food and Agriculture Organization of the United Nations (FAO), livestock contributes 40% of the global value of agricultural output and supports the livelihoods and food and nutritional security of almost 1.3 billion people [10]. BSAAOs promote healthy soil function and structure. Poultry litter and animal manure provide sources of nitrogen, phosphorous, and secondary nutrients like calcium, magnesium, and sulfur, along with micronutrients important for crop production [9,33,34]. Organic material provided by BSAAOs enhances the density, structure, and water-holding capacity of the soils, thereby increasing crop yield [35,36]. Although there are numerous benefits to soil and crops, BSAAOs, whether raw or treated, increase the risk for crop contamination via zoonotic bacteria, protozoa, and viruses.

Treated BSAAOs also pose a risk for pathogen survival and crop contamination. Xiong et al. [37] evaluated the effect of organic fertilizers on the survival of non-pathogenic and pathogenic *E. coli* in soils and their transfer to lettuce. Biological soil amendments are an essential input in organic lettuce production. However, these organic fertilizers can introduce or transfer pathogens like *Escherichia coli* O157:H7 to lettuce in pre-harvest environments. In this study, the soil was amended (side-dressed) with either heat-treated poultry pellets (HTPPs), HTPPs with corn steep liquor (CSL), seabird guano (SBG), SBG with CSL, or left unamended (UA). The Romaine lettuce was harvested after 28 days, and the presence of *E. coli* was determined. After 28 days, it was found that 13.3% (6/45) and 11.1% (5/45) of lettuce plants contained non-pathogenic and pathogenic *E. coli*, respectively [37]. These data shows that treated biological amendments used in organic lettuce production have the potential to assist *E. coli* survival in soil.

A study conducted by Nguyen et al. [38] determined that the microbial risks associated with liquid-based fertilizers in combination with treated BSAAOs may differ in organic lettuce production in the southwest desert. Despite treatment, BSAAOs can support the growth and survival of pathogenic *E. coli* in leafy green production. Additionally, there is a growing demand for non-BSAAO sugar-based organic liquid fertilizers (for example, corn steep liquor) with unknown microbial food safety implications. Corn steep liquor (CSL) treatments and heat-treated poultry pellets (HTPPs) supported higher levels of *E. coli* on the lettuce plants compared with seabird guano (SBG) pellets (*p* < 0.05) [38]. Increased *E. coli* reduction in soils correlated positively with higher soil temperatures and longer sunlight exposures during the growing season. Similarly, a 2-year (2018 and 2019) study conducted by Litt et al. [5] determined longer survival duration in soils with treated BSAAOs. The transfer of *E. coli* to cucumbers from inoculated BSAAOs, such as HTPP-amended soils, was evaluated. In 2018, *E. coli* was recovered from 97.5% of the cucumbers sampled, with levels ranging from 1.23 to 6.03 log most probable number (MPN)/gm. In 2019, 35% of the cucumbers tested contained *E. coli* with levels ranging from 0.01 to 2.63 log MPN/gm [5]. This shows that pathogens can persist on treated BSAAOs and potentially pose a risk for contamination.

Additionally, Kharel et al. [39] evaluated the effect of agricultural soils amended with HTPPs and composted poultry litter (PL) on *E. coli* survival in south-eastern soils (Florida and Georgia) over 140 days. Raised bed plots were left unamended (UN) or amended with PL or HTPPs (680 g/plot). Each plot was spray-inoculated with 1 L of rifampicin-resistant *E. coli* (7–8 log CFU/mL). In Florida, HTPP-amended soils supported higher levels of *E. coli* compared to PL-amended soils; in Georgia, similar survival was observed between PL- and HTPP-amended soils. This study highlights that in the southeast US, the use of BSAAOs in soils can prolong the survival of *E. coli* irrespective of factors intrinsic to the location and have implications regarding food safety practices [39].

Bacterial and viral pathogen persistence in raw animal manure is dependent on various factors such as manure type, method of application, method of incorporation, soil type, storage of manure prior to application, and the current microbial diversity within the soil [40,41,42]. Geographical and environmental factors also greatly influence bacterial survival [5]. Examining the survival of pathogens in manure is important, but focusing on the survival of enteric pathogens in manure once they have been applied and incorporated into the soil as fertilizer or organic amendment can influence or support food safety practices. Pathogens survive differently across different amendments when compared to soils that contain those amendments [43].

Several published field trials have previously examined the use of treated versus untreated animal manure over time. In the mid-Atlantic U.S., specifically, fields located in Maryland and Pennsylvania, twelve separate field trials were conducted over a four-year period at three different farms to evaluate the survival of a multi-strain inoculum of *E. coli*, including both non-pathogenic and attenuated-O157 [11]. The soils were inoculated with high and low levels and amended with either poultry litter, dairy cattle manure, horse manure, or left unamended with surface application or tilled into soils. This study determined that several spatiotemporal factors, such as season, site, and year, affect the survival and persistence of *E. coli* in manure-amended soils when compared to agricultural factors such as manure type, depth of application, and organic or conventional management of soils. *E. coli* survived for a maximum of 219 days throughout the studies and ranged from 0.14 to 2.32 log CFU/g soil, depending on the initial inoculum levels, amendment type, and depth of application [11]. Weather, such as rainfall and daily average temperature, also impacted *E. coli* survival and persistence, more so than agriculture effects. Across the multi-year studies, it was determined that poultry litter supported longer survival of *E. coli* when compared to other manure types in 60% of the studies conducted [11]. Pang et al. [44] used data from this study and found that days post inoculation, inoculum level, amendment type, days of rain since previous sampling day, and soil moisture content were the five most influential factors in predicting the quantitative levels of *E. coli*. They also determined that poultry litter supported the highest quantitative levels of *E. coli* when compared to dairy manure, horse manure, or no amendment [44]. A study conducted by Dunn et al. [45] evaluated prevalence and concentration of *Salmonella enterica* in poultry litter in the southern United States. Poultry litter from thirteen farms across four southern states was examined for *Salmonella*. A total of 33 samples out of 490 (6.7%) tested positive for *Salmonella* [45]. The concentrations of *Salmonella* in contaminated poultry litter were variable, ranging from <0.45 to >280,000 MPN/g [45]. The most prevalent serotypes found were Kentucky (45.5%), Kiambu (18.2%), and Michigan (12.1%). *Salmonella* Kentucky also had the greatest distribution and was found on four of the six (66.7%) positive farms [45]. The results from this study demonstrated that *Salmonella*’s prevalence and concentration in poultry are variable. Maintaining good agricultural practices is critical when using poultry litter as a soil amendment on fresh produce because *Salmonella* can survive and cross-contamination can occur. Another study by Çekiç et al. [46] evaluated the influence that spatial and temporal factors have on the persistence of non-pathogenic populations of *E. coli* in cattle manure-amended soils. This study showed that different sites did not influence survival rates; however, time after application, depth, and manure application rate did have an effect. Increased manure applications, which potentially could have contained higher *E. coli* populations, supported longer survival durations when compared to lower applications. This study is unique in its determination of *E. coli* survival, which was determined to be of greater duration in the fall seasons, lasting 252–280 days, when compared to the summer season where *E. coli* was detected as lasting 56–112 days. The soil and air temperatures were significantly lower in the fall (17–19 °C) compared to the summer (26–27 °C) [46].

It is critical to evaluate the survival of pathogens in BSAAOs because of the longer survival duration at higher levels and the increased likelihood of transfer of the pathogens to the edible portion of the crop. A study was conducted in the mid-Atlantic area of the United States in clay-loam soils, where over the course of two seasons, *E. coli* persistence was monitored in soils containing either poultry litter, composted poultry litter, or heat-treated poultry litter pellets along with their transfer to growing cucumber crops in the field covered with either plastic mulch or left uncovered [5]. During the study, physicochemical and nutrient measurements were analyzed for their ability to predict *E. coli* levels in BSAAO-amended soils. Nitrate levels and moisture content were determined to be strong predictors of *E. coli* survival in soils. However, upon further analysis, it was determined that the evaluation of the combination year, amendment, and mulch type produced a better predictor for *E. coli* survival in soils where cucumber plants were grown than any single nutrient or physical measurement [5]. A longer persistence of *E. coli* and more transfer of *E. coli* from unamended or amended soils to cucumber plants and fruit were observed in year 1 (77 ± 30 days) to achieve a 4 log CFU/g reduction, compared to year 2 (56 ± 21 day) [5]. The cumulative rainfall observed in year 1 was approximately 24.94 inches (63.34 cm), which was nearly twice the amount that was observed in year 2, which was approximately 12.63 inches (32.08 cm). This could have influenced the survival durations and transfer to cucumbers [5]. In year 1, regardless of soil amendment treatment, 98% of cucumbers that were harvested were contaminated with *E. coli*. In year 2, *E. coli* was detected on only 35% of the cucumbers harvested, which highlights that *E. coli* survival was extended by the addition of several poultry litter-based soil amendments. These were commonly used in the organic production of fruits and vegetables and were highly dependent on temporal variation in rainfall [5].

Another study that evaluated a multi-season survival of *E. coli* in soils containing treated amendments showed that *E. coli* survived for longer durations in soils amended with poultry litter compost or heat-treated poultry pellets compared to soils amended with dairy manure compost [15]. The researchers determined that *E. coli* survived for longer durations in clay soil compared to sandy loam soil, with water potential and daily temperature impacting survival duration as well [15].

Treated BSAAOs have been known to reduce the risk of microbial contamination that can be introduced through direct application to soils; however, treated BSAAO-amended soils may also provide an opportunity for pathogens to persist, by providing enhanced nutrients when pathogen contaminants are introduced through contaminated irrigation water or animal intrusion [47]. A common environmental issue can include nutrient runoff from soils, which may then contaminate local waterways. Soil runoff containing BSAAOs may provide nutrients for pathogens in irrigation water sources. In one study, *Salmonella* Newport grew to higher populations in simulated runoff containing heat-treated poultry pellets [14]. Levels increased by 3–5 log CFU/mL compared to runoff that did not contain heat-treated poultry pellets, where the levels increased by 1–3 log CFU/mL. This study supports the hypothesis that heat-treated poultry pellet-amended runoff alleviated nutrient stress placed on *Salmonella* Newport. The *Salmonella* Newport strain did not possess the rpoS gene—which is the universal stress response gene that encodes a sigma factor called RpoS, also known as sigma-38—and was not able to grow in simulated soil runoff without heat-treated poultry pellets. In a study by Neher et al. [48], similar results were observed with non-pathogenic *E. coli* in poultry litter-based compost soil runoff and dairy manure-based compost soil runoff, where levels increased between 6 and 9 log CFU/mL. In both experiments, it was observed that the runoff contained fewer indigenous bacteria, which allowed for increased growth of *E. coli* and *Salmonella*, indicating that indigenous bacterial populations in soils may compete with enteric bacterial foodborne pathogens and limit their growth [48].

Overall, pathogen survival in BSAAOs is dependent on the pathogen, amendment type, temporal, weather, and environmental factors. Further research is needed to understand pathogen survival and persistent trends.

## 4. Adjacent Land Use and Risks of Windborne Transmission

The use and application of BSAAOs on adjacent lands is defined as within 500 feet or 152 m of agricultural land that is actively engaged in properly conducted agricultural operations [49], and the proximity of concentrated animal feeding operations (CAFOs) to produce fields can increase the potential risk of contamination [7]. The current guideline for crops adjacent and nearby land used for an animal feed operation (AFO) is 30 feet (9 m) if there is no composting in use and 400 feet (122 m) if there is composting in use [7]. The current LGMA standards state that a 1200 ft (366 m) distance must be maintained for <1000 head/cattle and 1 mile (1.6 km) for more than 80,000 head/cattle between a concentrated animal feeding operation (CAFO) and a field of leafy greens [7]. Although these guidelines are in place, contamination from raw manure can occur by contact with the field via windborne transmission from manure-contaminated dust particles [50]. Manure-contaminated dust particles can therefore come into contact with adjacent fields, produce, or agricultural water [50].

Dust containing soil and manure particles may contain pathogens such as *E. coli* O157:H7 or *Salmonella* and movement of the cattle herds or other animals in the operation can cause dust or manure to become airborne and subsequently contaminate crops. One multi-season study showed that spinach, mustard, and turnip greens planted adjacent to a cattle feedlot were contaminated with pathogenic *E. coli* O157:H7 that originated from the cattle feedlot [51]. A proportion (1.8%) of leafy green samples that were planted 180 m (590 ft) away from the feedlot were positive for *E. coli* O157:H7, which was a significantly lower proportion than that found (3.5%) in leafy greens planted closer, at 60 m (197 ft) away [51]. A study conducted by Nguyen et al. [52] evaluated the survival of *E. coli* in airborne and settled poultry litter. To determine the survivability of airborne *E. coli*, an AGI-30 bioaerosol sampler (AGI-30) was used to collect the *E. coli* at 0 and 20 min after aerosolization. The results showed that the half-life time of airborne *E. coli* was 5.7 ± 1.2 min [52]. The survivability of *E. coli* in poultry litter and settled *E. coli* was much longer with the half-life times of 15.9 ± 1.3 h and 9.6 ± 1.6 h, respectively [52]. In addition, the size distribution of airborne *E. coli* attached to dust particles and the size distribution of airborne dust particles was determined to be important. Dust particles larger than 2.1 mm in diameter were able to carry *E. coli* farther and survive longer. This study explains the risk that land-adjacent poultry farms pose to fresh produce as contamination via fugitive dust is possible.

Studies have shown that *Salmonella* spp. can survive in turkey manure dust and their survival rate is dependent on soil moisture and dust particle size [50]. One study observed that a multi-strain inoculum of *Salmonella* survived for more than 290 days in manure dust with a particle size of 125 µm and with a moisture content of 5% [50]. Which was a longer period when compared to larger particle sizes of 212 µm, 355 µm, and 500 µm, and compared to higher moisture contents of 10% and 15% [50]. Although manure dust can transport pathogens longer distances of up to 400 ft (122 m) [51], it likely also allows for persistence and prolonged survival of bacterial pathogens on leafy green foliar surfaces [50]. Environmental factors, such as wind speed or dry conditions, along with adjacent land use issues such as the application of BSAAOs to fields adjacent to growing crops, can influence the transfer of contaminated dust containing foodborne pathogens.

### Case Studies of Windborne Transmission

In 2018, in the Yuma, Arizona, growing region, an outbreak of gastroenteritis associated with the *E. coli* O157:H7 contamination of Romaine lettuce occurred. This was an outbreak across 36 states, infecting a total of 210 people, and caused 96 hospitalizations, including 27 people who developed hemolytic uremic syndrome [53]. Five deaths were reported in Arkansas, California, Minnesota (2), and New York. Windborne transmission of contaminated dust from the adjacent CAFOs onto Romaine lettuce crops or canal water was assessed in the environmental investigation following the outbreak [54]. The CDC laboratory tests identified the outbreak strain of *E. coli* O157:H7 in water samples taken from an irrigation canal in the Wellton area, near the Yuma, AZ, produce-growing region and the strain was genetically similar to environmental isolates of *E. coli* O157:H7 found at an adjacent cattle ranch, also referred to as a CAFO [54]. A traceback of the Romaine lettuce that was consumed by infected individuals determined that the origin of the outbreak was from the Yuma produce-growing region [54]. The traceback identified a total of 36 fields in 23 farms in the Yuma growing region as potentially supplying the contaminated Romaine lettuce which was consumed and linked to the outbreak [54]. Since these isolates were detected during the irrigation of canal water, windborne *E. coli* O157:H7 could have been deposited onto the Romaine lettuce from the adjacent cattle farm, which could be speculated to be the source of contamination [54].

Additionally, another multistate outbreak involving a *Salmonella* Newport contamination of red onions occurred in 2020 in California, involving potential windborne dust contamination. There were 1127 confirmed cases across 46 states [55], 167 people were hospitalized and no deaths were reported [55]. Epidemiological and traceback investigations conducted by the Public Health Agency of Canada (PHAC) and The Canadian Food Inspection Agency (CFIA) identified red onions from Thomson International Inc. as the source of this outbreak [55]. It was also found that other types of onions (such as white, yellow, and sweet yellow) were also contaminated with *Salmonella* Newport as they were also grown, harvested, and processed alongside the originally contaminated red onions [55]. On 1 August 2020, Thomson International Inc. recalled all red, yellow, white, and sweet yellow onions because there was potential contamination with *Salmonella* [55]. FDA’s traceback investigation identified a packing facility and multiple farms that supplied red onions that could have been potential sources of contamination [55]. Field-level investigations were conducted after identifying the suspect vehicle and firm within Thomson International Inc.; however, the field investigations were limited since most of the onions had already been harvested and distributed. The FDA completed over 2000 product and environmental analyses from multiple Thomson International Inc. locations and surrounding areas which include water, soil, and scat samples [55]. A conclusive root cause could not be identified for this outbreak; however, several potential contributing factors to this outbreak were identified, including a leading hypothesis that potential windborne contamination of irrigation water from a nearby poultry farm that was used in a growing field in Holtville, California, may have led to the contamination of the red onions [56]. During a follow-up investigation, peach orchards adjacent to a cattle feedlot and poultry farm contained *Salmonella* Montevideo, which was genetically similar to the 2018–2020 beef and cattle isolates, and the peach tree leaves contained *Salmonella* Alachua [57]. These samples were collected near the adjacent poultry farm which reported genetically similar isolates to 2019 and 2020 chicken isolates [57]. While none of the environmental *Salmonella* isolates recovered were the outbreak *Salmonella* Enteritidis isolate, these results show that fugitive dust from adjacent agricultural operations can impact produce farms and can provide a potential route for the contamination of produce grown on adjacent or nearby lands [10]. Windborne transmission of pathogens via adjacent lands within close proximity to CAFOs and AFOs is an important risk factor to consider. There is a need for a novel pre-harvest food safety approach to address concerns revolving around adjacent land use and contaminated manure or the risks of using untreated BSAAOs.

## 5. Soil Amendment Remediation

Soil amendments have become more widely useful for remediation purposes. Soil contamination by potentially toxic elements (PTEs) could lead to adverse environmental impacts. Using soil amendments to reduce waste and PTEs could be an efficient and effective control method. Increased poultry and livestock production has also increased waste products that are land-applied to improve the quality of contaminated soils [58]. Soil amendments such as manure, compost, biochar, clay minerals, phosphate compounds, coal fly ash, and liming materials (acid-soluble materials applied to soils primarily to raise the pH of acidic soils, such as carbonates, oxides, and hydroxides of Ca and Mg) are assessed for their ability to immobilize PTEs [58]. For example, the use of biochar, a solid material obtained from the thermochemical conversion of biomass in an oxygen-limited environment, can be utilized in carbon sequestration, carbon farming, climate change mitigation, soil pollution remediation, and bioremediation [59].

Biochar can increase soil nutrient availability, microbial activity, soil organic matter, water retention, and crop yields in soils [59]. The interaction of biochar with compost and other soil amendments has been associated with a high adsorption of several pollutants [59]. One study evaluated biochar filters as an on-farm treatment to reduce pathogens when irrigating with wastewater-polluted sources [60]. The biochar was successful at filtering and removing *Saccharomyces cerevisiae* (>1 log CFU) from diluted wastewater; however, it was unsuccessful at removing bacteria (*E. coli* and *Enterococcus* spp.) and viruses (bacteriophages MS2 and ϕX174) (~1 log CFU or PFU). It was suggested that particle diameter of the biochar was the main explanation for the variation in microbe removal. In a review by Enaime et al. [61], it was suggested that pyrolysis temperature can play a huge factor in biochar’s absorption. For example, pyrolysis at high temperatures resulted in hydrophobic biochar with a higher surface area and micropore volume, allowing it to be more suitable for organic contaminant absorption [61]. Biochar produced at low pyrolysis temperatures resulted in smaller pore sizes, lower surface areas, and higher oxygen-containing functional groups, which are more suitable for removing inorganic contaminants [61].

Overall, like any novel technology, further research is needed to determine how different factors might influence the capacity of biochar to absorb pollutants and act as a bioremediation technology for pathogens in agricultural soils. Existing studies are generally in agreement in their conclusions, in that utilizing biochar to amend agricultural soils has value as a means to reduce microbial contaminant levels and improve soil health. The promising results from studies centered on biochar also emphasize the untapped potential of other novel bioremediation technologies such as fungal biocontrol or mycoremediation.

## 6. Fungal Biocontrol

Bioremediation techniques have been used over the years to remove heavy metals from contaminated soil and chemically breakdown pesticides associated with industrial use; however, through the novel use of biocontrol agents, the bioremediation of manure is an emerging field of research. The risks of pathogenic contamination of biological soil amendments could be reduced by developing a cost-effective and sustainable mycofiltration practice using naturally formulated by-products from filamentous fungi. Of the many microbes used to remediate contaminated sites, ligninolytic white-rot fungi (WRF) can degrade a wide variety of toxic or persistent environmental contaminants [62]. White-rot fungi are capable of detoxifying and degrading pollutants in the environment such as polycyclic aromatic hydrocarbons (PAHs) [63], pentachlorophenol (PCP) [64], carbamazepine [65], estrogen compounds [66], atrazine [67], and alachlor [68]. The chemical structure similarities between lignin and many recalcitrant contaminants make white-rot fungi the ideal candidate for the remediation of these pollutants [69].

As novel biocontrol agents are increasingly explored, bioremediation strategies of manure are also becoming an emerging field of research. The risks of transfer of contaminated biological soil amendments to edible portions of crops could be reduced by developing a cost-effective and sustainable mycofiltration/mycoremediation practice by using naturally formulated by-products from filamentous fungi in soil amendments. By using naturally formulated fungi, growers would be able to still utilize untreated BSAAOs while also limiting the risks associated and maintaining low economic costs.

### 6.1. White-Rot Fungi

White-rot fungi are a physiological grouping of fungi within the Basidiomycota phylum and have the unique ability to completely degrade lignin in the environment [70]. Lignin is the major structural component of woody tissue along with hemicellulose and cellulose. Lignin is a complex polymer consisting of phenyl propanoid units linked by various carbon–carbon and ether bonds. Lignin is slowly degraded in the environment by the involvement of bacteria, actinomycetes, yeasts, and fungi [71]. The fungal degradation of lignin is well studied and usually classified by the type of decay produced. Wood-rotting fungi can be classified as soft-rot, brown-rot, or white-rot fungi. Soft-rot decay causes cavities in the cell wall of moist wood and is caused by molds of ascomycetes and Imperfect Fungi. They mainly degrade the cellulose through hyphae penetration and the secretion of enzymes. However, degradation of hemicellulose and lignin has been shown to occur to some extent [72]. Brown-rot fungi are the most common in soft wood and include several species of basidiomycetes. They degrade the cellulose and hemicellulose, and they use a non-enzymatic process to depolymerize lignin, which repolymerizes, resulting in a brown friable residue creating the visible brown rot [71,72].

White-rot fungi are unique from their brown-rot and soft-rot fungi counterparts in that they completely mineralize lignin and carbohydrate compounds in wood to CO_2_ and water. Between 1600 and 1700 species of basidiomycetes and a few species of ascomycetes can mineralize lignin by using a complex, hydrogen peroxide-dependent, oxidative, enzymatic, radical-mediated mechanism. However, the actual physiological process is specific for each fungus. The enzymes that are required to depolymerize lignin include lignin peroxidase (LiP), manganese peroxidase (MnP), laccase, versatile peroxidase, and other hydrogen peroxide-generating enzymes [73]. Evaluating white-rot fungi’s use in biofiltration systems—which are systems where organic, porous materials like sand or even fungal mycelial mats can serve as the filter medium and microorganisms carry out treatments—may present a potential pre-harvest approach for inactivating pathogenic material. In biotechnological industries as well as wastewater processing technologies, bioreactor systems, which may include fungal systems, continue to be optimized, including study of use of enzyme production and pollutant-degrading microorganisms [74,75,76]. Within the field of environmental engineering, these fungal reactors have been used for bioremediation practices to aid in effectively breaking down contaminants and persistent toxic chemicals [75,76]. Previous studies have shown a significant reduction in various *E. coli* concentrations in wastewater and stormwater runoff through the use of filamentous fungi [77]. Fungal bioreactor systems have the potential to alleviate pathogen load in wastewater, stormwater, and agricultural runoff in a similar means to that of dairy manure, as was shown by Chirnside et al. [77]. A 2 log reduction was observed in *E. coli* in food processing wastewater through the use of white-rot fungal species, *Pleurotus ostreatus*, grown on woodchips [77]. Taylor et al. [21] found that *Stropharia rugosannulta*, an edible fungus characterized as a litter-decomposing fungus, grown in an alder woodchip bioreactor, was able to reduce *E. coli* by 22% in synthetic wastewater. While Umstead [78] found a 100% removal of *E. coli* K12 in synthetic wastewater by *Pleurotus eryngii*, *Piptoporus betulinus*, and *Daedaleopsis congragosa* grown in separate woodchip bioreactors.

### 6.2. Ligninolytic Activity of White-Rot Fungi

Fungi have various profiles of production of different ligninolytic enzymes. To break down lignin, most white-rot fungi produce three extracellular lignin-modifying enzymes: LiP, MnP, and laccase (Lac) [79,80]. These lignin-modifying enzymes oxidize and mineralize their substrate whether the substrate is lignin or a recalcitrant chemical, decomposing it into an available form for plants [81,82]. When white-rot fungi are in the active phase of secreting these three enzymes, the fungi are described to be in ligninolytic activity. Ligninolytic activity allows the fungus to inhibit and degrade a wide range of materials, such as bacteria and nematodes, because the fungus is secreting ligninolytic enzymes [83]. Ligninolytic activity is triggered by nutrient-limiting conditions or by ‘starving’ the fungi. Predatory behaviors, as well as the ligninolytic activity of the fungi, can be activated when key nutrients, such as nitrogen and phosphorus, are limited [84]. The induction of ligninolytic activity was thought to be idiophasic or part of secondary metabolism. The induction of LiP and MnP activity was achieved by stressing the cultures by nutrient starvation (either carbon or nitrogen) or by exposing the system to high oxygen concentrations [85]. Laboratory studies also found that the activation and mechanistic pathway of ligninolysis was affected by oxygen tension, carbon substrate, nutrient nitrogen, H_2_O_2_, pH, and cultural agitation. Because of the differences found in lignin degradation among white-rot fungi, researchers have found that the stimulation of ligninolytic activity is determined by the fungal species, the mechanism involved, and the environment of the system [85]. Through ligninolytic activity, fungi may produce co-substrates and chelators that are important in enzyme activation, and these compounds have been studied for their influence on induction of ligninolytic activity.

The process and rate at which the white-rot fungi degrade lignin and other carbohydrate compounds can also differ among species. Typically, two separate decay patterns are used. The first pattern is a nonselective degradation of all wall components. The second pattern is the selective degradation of lignin and hemicellulose, which results in cellulose microfibrils remaining. *Phanerochaete* species have been shown to use the second pattern and the *Pleurotus* species delignifies wood by preferentially attacking the lignin more readily than the cellulose and hemicellulose, leaving enriched cellulose [86].

It also has been suggested that white-rot fungi may break down various nutrient sources, such as nematodes and bacteria, through the use of mycotoxin. To immobilize its prey, some species of white-rot fungi can secrete a mycotoxin, called ostreatin, and its hyphae will infiltrate its prey and absorb the nutrients [83]. Recently, it has been suggested that ostreatin, produced by some *Pleurotus* species of white-rot fungi and by other basidiomycetes, could be a ribotoxin-like protein [87]. A group of specialized ribonucleases found in basidiomycetes known as ribotoxin-like proteins (RL-Ps) can catalyze the endonucleolytic cleavage of large ribosomal RNA at a precise location within the sarcin–ricin loop (SRL) [88]. Ribotoxin-like proteins can be described as specialized ribonucleases found in mushrooms that selectively cleave a single phosphodiester bond within the SRL of the large ribosomal RNA [88]. When the SRL is cleaved, this can alter how it interacts with specific ribosomal proteins, for example, the P-stalk [88]. This disruption will then stop protein synthesis and the damaged ribosomes are prevented from interacting with additional elongation factors [88]. This could be one way that ostreatin allows the *Pleurotus* species to immobilize its prey.

The use of the mycotoxin ostreatin to break down nutrients in the environment is very unique and not all white-rot fungi have this capability. The production of lignin-degrading enzymes is more widely studied for mycoremediation and bioremediation. Two species of white-rot fungi that have been well studied throughout the literature for bioremediation purposes because of their ligninolytic activity capabilities include *P. ostreatus* and *Phanerochaete chrysosporium*.

*P. ostreatus* is a species of white-rot fungi that can break down recalcitrant compounds and is capable of bioremediation through the production of lignin-modifying enzymes. *Pleurotus* spp. are thought to degrade lignin using laccases, versatile peroxidases (VPs) and MnP enzymes. The study of other white-rot fungi has found other enzymes involved in lignin degradation, such as dye-decoloring peroxidases. How these enzymes are utilized and in what combinations varies with the particular species of white-rot fungi [86].

One of the first studies that evaluated *P. ostreatus*’ ability to produce ligninolytic enzymes was conducted by Barron [89]. Although the enzymes were not identified in this early study, Barron [89] was able to determine that when *P. ostreatus* undergoes nutrient-limiting conditions, it will prey on other nutrient sources such as nematodes and bacteria, which were explored throughout this study. Although Barron [89] did not explore the ligninolytic enzymes being produced by *P. ostreatus*, there are several studies that do.

Mikiashivili et al. [90] evaluated the effects that carbon and nitrogen sources had on *P. ostreatus*’ ligninolytic enzyme activity. It was determined that in the absence of carbon and nitrogen sources, the ligninolytic enzyme’s laccase, manganese-dependent peroxidase (MnP), and peroxidase activity increased [90]. A review by Knop et al. [91] evaluated the *Pleurotus* genus and its ability to produce ligninolytic peroxidases. The lignin-degradation systems of many white-rot fungi, including the *Pleurotus* genus, comprise a triad on laccases, and two hemeperoxidases: LiP and MnP. The fourth player in the lignin biodegradation system is versatile peroxidase (VP). This review also suggested that MnP, VP, and laccase are the major parts of the *Pleurotus* ligninolytic system [91]. A study by Ergun and Urek [92] evaluated the use of solid-state fermentation on *P. ostreatus* to produce ligninolytic enzymes. The use of solid-state fermentation induced a low-nutrient environment for *P. ostreatus*, which allowed for higher yields of the enzymes evaluated in this study, such as laccase, MnP, and LiP [92]. In a study by Li et al. [93] the ligninolytic characteristics of *P. ostreatus* were evaluated while *P. ostreatus* was cultivated on cotton stalk media. This study measured different levels of LaC, MnP, and H_2_O_2_ as *P. ostreatus* broke down the cotton stalk. It was shown that LaC and MnP were all secreted in high amounts as the fungi was actively degrading the lignin in the cotton stalk, suggesting that *P. ostreatus* selectively secretes ligninolytic enzymes depending on the stage of degradation and whether the substance can be easily degraded [93]. Another study by Hewage et al. [94] evaluated spawn-based pellets of *P. ostreatus* as an applied approach to increase the production of laccase in water. The introduction of lignin to the *P. ostreatus* pellets in water, induced laccase production along with limiting carbon and nitrogen. This suggests that ligninolytic enzyme production can be induced through limiting essential nutrients but also by introducing lignin to help trigger enzymatic production [94]. *P. chrysosporium*, is another white-rot fungal species that is commonly selected for mycoremediation because of its unique ability to produce ligninolytic enzymes. The lignin-degrading process of *P. chrysosporium* has been studied extensively and involves the production of LiP, MnP, and hydrogen peroxide (H_2_O_2_), which are required to activate the enzymes. Ligninolytic activity also produces co-substrates and chelators that are important in the enzyme-activation process and the production of H_2_O_2_ [85]. The activation of LiP produces a porphyrin cation radical that can oxidize non-phenolic compounds twice before it returns to its resting state. MnP is dependent on Mn^2+^ for activation and catalyzes the oxidation of various phenolic compounds. During ligninolytic activity, *P. chrysosporium* also produces glyoxal oxidase (Glox), which oxidizes aldehydes to carboxylic acids, producing H_2_O_2_.

One of the first studies which evaluated *P. chrysosporium*’s ability to produce ligninolytic enzymes was by Keyser et al. [95]. This study was able to confirm that when *P. chrysosporium* inhabits a nitrogen-limited environment, it will produce ligninolytic activity and produce a variety of ligninolytic enzymes [95]. Specific enzyme production was not explored during this study; however, there are several studies that confirm enzymatic production. One study evaluated the stimulation of ligninolytic production of *P. chrysosporium* by using polyoxyalkanes [96]. It was reported that LiP activity and MnP activity increased after *P. chrysosporium* was introduced to certain polymers [96]. This indicates that certain polymers can stimulate ligninolytic activity in *P. chrysosporium*. Not only can polymers affect enzyme production, but culture conditions can also affect the production of ligninolytic enzymes and the separation of lignin peroxidase. A study by Wang et al. [97] worked on optimizing culture conditions in order to gain the highest yield of enzyme production. It was determined that a 60% salt precipitation (NH_4_)SO_4_ yielded the best results for LiP. MnP reached peak yield through the use of Kirk’s culture medium [97]. Another study explored ligninolytic production with solid-state cultures of *P. chrysosporium* through the use of different agro-industrial wastes [98]. LiP production was attained through the use of pulp and paper waste, manganese peroxidase (MnP) activity increased when veratryl alcohol and Tween-80 were included in various wastes and laccase production was obtained using brewery by-products, fruit processing, effluents from the pulp and paper industry, and marine based by-products [98].

In more recent studies, *P. chrysosporium* has been studied to determine LiP decolorization functions in melanin decolorizing extracellular peroxidase [99]. There are few enzymes known to properly decolorize melanin using LiP from lignin-degrading fungus, one example is *P. chrysosporium*, which is the most extensively studied. This study explored that while LiP and MnP can be produced to decolorize melanin, it was also determined that when LiP production is reduced, only within the presence of Mn^2+^, then MnP production is induced to decolorize melanin. This correlating response suggests that LiP may be an initial response enzyme and MnP could be a secondary response enzyme, as when one decreases, the other increases [99]. Another study evaluated the white-rot fungi, *P. chrysosporium*’s biodegradation of benzol[a]pyrene (BaP) through the use of ligninolytic enzymes LiP, MnP, and laccase [100]. It was suggested that LiP, MnP, and laccase could degrade BaPs through the oxidative activity of polycyclic aromatic hydrocarbons and mediated hydroxylation [100]. The addition of Tween-80 and polyethylene glycol monododecyl ether (Brij30) helped initiate biodegradation [100].

One study explored co-cultivation *P. ostreatus* and *P. chrysosporium* and their production of ligninolytic enzymes for dye decolorization under solid-state fermentation [101]. Both fungi were inoculated onto nitrogen- and carbon-limited media to promote the production of Lip, MnP, and laccase. High yields of LiP (656 U/g), MnP (982 U/g), and laccase (772 U/g) were produced, highlighting that the optimization of lignocellulosic waste decomposition in nutrient-limiting environments can produce high yields of ligninolytic enzymes, which could be further exploited for the decolorization of synthetic dyes.

### 6.3. Inhibitory Effects of White-Rot Fungi

White-rot fungi can be cultivated on certain substrates like woodchips, spent mushroom compost, and reticulated polyurethane foam to induce nutrient-limited conditions that trigger their ligninolytic activity [102]. Once the fungus is in ligninolytic activity, it will begin to produce ligninolytic enzymes which can be used to break down or decompose specific pollutants in the environment. Barron [89] has reported that when certain species of white-rot fungi are deprived of nutrients or experience nutrient limitations, they exhibit predatory behavior by extending their hyphae as a way to seek out alternative nutrients. When introduced to novel nutrient sources like bacteria, certain species of fungi will enter ligninolytic activity and begin to produce ligninolytic enzymes. The hyphae initiate the secretion of diverse ligninolytic enzymes to degrade bacterial cell walls and assimilate nutrients [89]. When *P. ostreatus* was grown on water agar plates inoculated with *E. coli*, it was observed to infiltrate and completely consume bacterial colonies within 72 h [77]. These studies suggest that white-rot fungi species have bioremediation potential, and the ligninolytic enzyme’s degradation capabilities should be explored.

One study evaluated six white-rot-fungal pretreatments, including *P. ostreatus* and *P. chrysosporium*, and they were used as a pretreatment on lignocellulosic biomass, corn stover, and their production of ligninolytic enzyme [103]. Over a 30-day cultivation period, the biomass degradation, enzyme production, hydrolysis-derived reducing sugars, and ethanol yield from yeast fermentation were quantified, with samples collected at five-day intervals. In this study, it was determined that fungal pretreatments enhanced the synthesis of a range of bioproducts, including enzymes, sugars, and ethanol, as well as lignin degradation by white-rot fungi to aid in biomass degradation [103].

In a review by Bautista-Zamudio et al. [104] the biodegradation of plastics by white-rot fungi was explored. One section of this review explored the biodegradation capabilities of *P. ostreatus* and *P. chrysosporium* on polyethylene. Polyethylene is a versatile thermoplastic found in a broad range of consumer goods, including milk bags, shopping bags, food packaging, laminates, and other everyday items. The degradation of polyethylene can be assessed by evaluating the polymer’s weight loss following exposure to white-rot fungi. *P. ostreatus* and *P. chrysosporium* showed the highest degradation under specific nutrient conditions. Another section in this review explored oxo-biodegradable plastic degradation. Here, it was observed that when carbon or nitrogen is removed, *P. ostreatus* exploits the plastic as a carbon source and begins to break it down for energy and nutrients [104].

In another review, species of white-rot fungi were explored as tools for the bioremediation of xenobiotics [105]. This review discussed the significant applications of ligninolytic enzymes in biotechnology, including laccase, MnP, and LiP, noting their broad substrate specificity which enables them to degrade a wide array of xenobiotic compounds, including synthetic dyes, chlorinated phenolics, pesticides, polycyclic aromatic hydrocarbons, chlorophenols, nitroaromatics, and explosives. On a more topical note, they also have the potential to contribute to the breakdown of novel contaminants like pharmaceutical residues, flame retardants, and PFASs. Laccase’s, MnP’s, and LiP’s degradation abilities are already applied in several biotechnological areas, including the bioremediation and decolorization of industrial effluents originating from textiles manufacturing, distilleries, pulp and paper production, and various wastewater sources, as previously discussed in this review; therefore, applying laccase, MnP, and LiP in the biodegradation of xenobiotic compounds is proposed to be the next step.

This review also explored bioremediation through the use of white-rot fungi. Bioremediation methods are utilized to remediate contaminated wastewater, soil air, and sediments where pollutants threaten ecosystems, animal health, or human well-being [105]. In a review by Hussain et al. [106], bioremediation with fungi, also known as mycoremediation, was explored. Both mycoremediation and bioremediation processes may be implemented at the contamination site (in situ) or at a separate location (ex situ). Approaches to in situ bioremediation focus on managing pollutants within the affected area, and may include methods like natural attenuation, bioaugmentation, bioventing, bioslurping, and biosparging [106]. A study conducted by Roy et al. [107] highlights that such techniques have demonstrated success in remediating environments polluted with chlorinated solvents, dyes, heavy metals, and hydrocarbons. Unlike in situ, ex situ bioremediation entails the removal of contaminated materials and relocating them for off-site remediation [105]. The application depends on whether contaminants can be extracted and transported to appropriate treatment facilities, including bioreactors, specialized landfills, and combined solid and liquid waste facilities. Conducting mycoremediation ex situ enables the enhanced regulation of environmental conditions and fungal development [105]. White-rot fungi can be utilized in fungal bioremediation through either fungal biomass or isolated enzymes. Fungal biomass within bioreactors can be utilized as pellets or immobilized on various solid support matrices, as was explored by Rodriguez-Couto [108]. The application of fungal biomass and enzymes in bioremediation offers a targeted approach to restoring polluted ecosystems and is a novel concept. Matrices need to be explored further to determine their nutrient-limiting capabilities to optimize ligninolytic enzyme production [105]. These studies and reviews suggest that further bioremediation implications of white-rot fungi via mycofiltration should be explored to determine their biodegradation effects on harmful pathogens.

One study by Liu et al. [109] explored *P. chrysosporium*’s ability to inhibit the pathogen *Fusarium pseudograminearum*, which predominately causes wheat crown rot in grain. This study observed that the mycelium of *P. chrysosporium* effectively inhibits the growth of *F. pseudograminearum* by tightly attaching to it and forming specialized penetrating structures. This process leads to the release of intracellular inclusions and the eventual disintegration of pathogen cells. This study did not focus on enzyme secretion; however, based on previous studies listed in this review, it could be theorized that ligninolytic enzymes (LiP, MnP, and laccase) were most likely secreted after *P. chrysosporium* mycelium penetrated *F. pseudograminearum* to aid in its degradation.

A study conducted by Omar et al. [110] analyzed the effects of *P. ostreatus* when grown on different matrices (including woodchips, spent mushroom compost, and foam) and the inhibitory abilities on *E. coli.* Lignin-degrading white-rot fungi has been known to reduce zoonotic bacteria in biological soil amendments of animal origin when included in a continuous flow-through system [110]. It was determined that *P. ostreatus* was able to inhibit *E. coli* across all matrices (*p* < 0.0001) and a greater reduction was witnessed in woodchips when compared to spent mushroom compost and reticulated polyurethane foam, indicative of the ligninolytic activity and overall fungal activity [110].

Another study by Omar et al. [111] evaluated the removal of pathogenic and non-pathogenic *E. coli* and *Salmonella* spp., utilizing the white-rot fungi species *P. chrysosporium* within a filtration system. Mycoremediation has been shown to increase microbial removal from water by filtration. The activation of white-rot fungi’s ligninolytic activity along with bacterial inhibition through the use of biochar and zero-valent iron (ZVI) within a filtration system, creates a novel filtration system to reduce pathogens from agricultural water [111]. It was determined that *P. chrysosporium* combined with BC and ZVI removed >4 log CFU/mL of *E. coli* and *Salmonella* from water, a useful filtration system for irrigation water [111]. This shows white-rot fungi’s potential for removing pathogenic bacteria from agricultural water.

Based on these findings, a more recent study evaluated the reduction in two *E. coli* strains (*E. coli* TVS355 and *E. coli* O157:H7 4407) in aqueous dairy manure and PBS in the presence of white-rot fungi *Pleurotus ostreatus* on three different nutrient sources (woodchips (WCs), spent mushroom compost (SMC), and reticulated polyurethane foam (RPF)) [112]. Overall, this study determined that *E. coli* TVS355 remained persistent in aqueous dairy manure and PBS and survived for 50 days in the presence of *P. ostreatus*, with a final concentration of 4 log CFU/g in aqueous manure and 7 log CFU/g in PBS [112]. However, a greater reduction in *E. coli* O157:H7 (*p* < 0.0001) was observed, as it survived for 50 days at an average of 4 log CFU/g in aqueous dairy manure and an average of 3 log CFU/g in PBS [112]. Therefore, the white-rot fungi, *P. ostreatus*, when grown on reticulated polyurethane foam, showed positive results as a potential nutrient-limiting resource. This approach could be the key to increased bacterial reductions, as the nutrient limitation drives increased ligninolytic activity to seek other nutrient sources [112].

## 7. Limitations of Using White-Rot Fungi for Bioremediation

The enzymatic machinery of white-rot fungi allows them to be attractive candidates for bio- and mycoremediation applications. In spite of their great mycoremediation potential, there are certain limitations and challenges that need to be addressed before this technology can be implemented at the industrial and commercial level. These challenges can include, but are not limited to, accurate selection of fungal strain, environmental factors that could affect bioremediation, and the stability and activity of ligninolytic enzymes.

There are an estimated 2–11 million fungal species in the world and only 155,000 have been named [113]. An area of research should focus on isolating new fungal strains with novel physiochemical characteristics. Another area of focus should be the isolation of indigenous fungal strains from polluted environments, since they could be adapted to high concentrations of pollutants and potentially could have evolved to express novel capabilities. Additionally, there are fungal taxa that have not been fully explored that may produce ligninolytic enzymes at higher yields and may be more stable under extreme environmental conditions such as pH or temperature. For example, aquatic fungi are extremely understudied. Bongugli-Santos explored the use of marine fungi isolated from marine sponges, and their potential ability to produce ligninolytic enzymes and decolorize Remazol Brillant Blue (RBBR) [114]. Some authors suggest that future research should aim to explore different fungal taxa to uncover fungal species that are effective bioremediating and mycoremediation agents.

Another limitation that effects the practical use of white-rot fungi bioreactor systems for the use of bioremediation is the fact that most fungal bioremediation research is still performed under ideal conditions. To determine whether or not fungal bioremediation is successful, there is a need to conduct studies under non-sterile conditions to evaluate various environmental factors and external pollutants’ effects on the degrading capabilities of these white-rot fungi. However, moving studies toward non-sterile conditions raises major concerns regarding nutrient availability for the fungi. Since the production of ligninolytic enzymes by white-rot fungi is induced by nutrient limitation and animal manure is rich with organic matter which can provide nutrients to the fungi, this may make bioremediation applications difficult. Furthermore, oxygen availability is critical for the growth of white-rot fungi, meaning that their ability to act as a bioremediation technology in low-oxygen environments, such as aqueous dairy manure, may be limited. Observing native bacterial and fungal competition is also important in order to evaluate the degradation efficiency of white-rot fungi.

Ligninolytic enzymes such as laccases, MnPs, and LiPs should be further investigated for higher yield rates and for the determination of whether ligninolytic enzymes are best suited for environmental bioremediation and mycoremediation practices.

## 8. Conclusions

Mycofiltration systems, which employ fungal networks to filter and remediate contaminated soil amendments or water, represent an innovative approach to bridging conventional treatment technologies with low-cost, biologically driven alternatives for pre-harvest food safety. A growing body of research has demonstrated that certain white-rot fungi possess the capacity to inhibit or reduce bacterial pathogens in wastewater [115]. Notably, *P. ostreatus* has been observed to exhibit predatory activity against generic *E. coli* [77]. These findings highlight the ecological potential of fungal–bacterial interactions, wherein fungal hyphae may immobilize, degrade, or consume bacteria, thus reducing pathogen loads in the selected environment. Such mechanisms suggest that white-rot fungi could serve as a biological agent for pathogen suppression, offering a natural complement to existing chemical or mechanical treatment methods.

Exploring novel mycofiltration systems bridges the gap between conventional technologies and low-cost treatment alternatives for pre-harvest food safety. Building upon these novel approaches, mycoremediation can be strategically incorporated into pre-harvest safety methods to facilitate the removal of pathogenic microorganisms. Bioreactors designed to harness the metabolic versatility of white-rot fungi provide a low-cost alternative to traditional treatment methods. Beyond pathogen reduction, mycofiltration systems offer additional benefits, including the potential to recycle organic matter, reduce reliance on energy-intensive or chemical treatments, and adapt to diverse agricultural contexts where resource efficiency is critical. Integrating fungal bioremediation into food safety management could not only innovate biological solutions but also contribute to broader goals of sustainability, resilience, and public health protection in agricultural systems.

## Figures and Tables

**Table 1:** Studies that use thermal, chemical, or biological treatments and their reported bacterial or viral reductions and survival post treatment.

Treatments	Treatment Process	Soil Amendments	Organism	Reported Reduction	Survival Duration	Reference
Thermal	Heat-treated	HTPPs *	*Salmonella* Newport	3–5 log CFU/g	>91 days	[14]
	Heat-treated	HTPPs *	*E. coli*	4 log CFU/g	>120 days	[5]
	Heat-treated	Dairy manure and poultry litter compost, HTPP *	*E. coli*	1.69 log CFU/g	>104 days	[15]
			*Listeria* spp.	Not detected	N/A	
Chemical	Ozonation	Swine manure	*E. coli*	3 log CFU/mL	>3 days	[16]
			Total coliforms	1 log CFU/mL	>3 days	
	Ca(OH)_2_ Phosphorus removal	Swine slurry	*E. coli*	1 log CFU/mL	3 h	[17]
			*Salmonella* spp.	1 log CFU/mL	3 h	
	NH_4_^+^ and NH_3_	HEAM **	Viruses	4 log PFU/mL	6 h	[18]
Biological	Conventional sludge treatment	Municipal sewage	*E. coli*	40 MPN g^−1^ d.m.	>60 days	[19]
			*Enterococcus* spp.	40 MPN g^−1^ d.m.	>60 days	
	Untreated	Aqueous dairy manure	Total coliforms	0.15–2.0 × 10^5^ L^−1^	>42 days	[20]
		Mycelial-infused woodchips	*E. coli*	0–3.5 × 10^5^ L^−1^	>42 days	
	Untreated		*E. coli*	20% reductions	30 min	[21]

* Heat-treated poultry pellets (HTPPs). ** Human excreta and animal manure (HEAM).

## Data Availability

No new data were created or analyzed in this study. Data sharing is not applicable to this article.
